# Analysis of fitness among Korean adults by the cause–effect relation in lump mean scheme

**DOI:** 10.1186/s13102-021-00338-5

**Published:** 2021-09-16

**Authors:** Nam Lyong Kang

**Affiliations:** Department of Nanomechatronics Engineering, Pusan National University, Busandaehang-ro 63 beon-gil 2, Geumjeong-gu, Busan, 46241 Republic of Korea

**Keywords:** BMI, Waist-to-height ratio, Obesity, Fitness, Lump mean value, Linearity, Gaussianity, cause-effect relation

## Abstract

**Background:**

The aim of this study was to examine whether the fitness of Korean adults can be analyzed by the cause-effect relation using the linearity or Gaussianity in the lump mean scheme (LMS).

**Methods:**

This study analyzed previous results for the sit-up test obtained in the LMS by regression analysis in Sigmaplot 14. The effects of the body mass index (BMI) and new waist-to-height ratio (WHT2R) introduced by the present author on fitness were investigated.

**Results:**

The distribution of the sit-up test score with respect to the BMI and WHT2R were interpreted by their Gaussianity and linearity, respectively. This means that the muscular endurance of males is determined by two causes (fat and muscle) when the BMI is a variable and one cause (abdominal fat) when the WHT2R is a variable.

**Conclusions:**

Personal exercise aims were simpler to establish using WHT2R than using BMI. On the other hand, it was recommended for people with a low BMI to increase their fitness using exercises that increase their muscle mass.

## Background

Despite their complexity, all phenomena (effects) in nature have causes, i.e., an effect cannot occur before its cause. An explicit description of the mechanism that generates a phenomenon can be obtained by knowing the reason for that phenomenon. Therefore, knowing the cause-effect relationships help understand how variations of certain variables (cause) influence other related variables (effects), which is important in various fields, such as medicine [1, 2], artificial intelligence [3, 4], natural science [5, 6], social science [7], and financial economics [8].

On the other hand, it is difficult to investigate a specific variable (cause) dependence on a multi-variable distribution (effect) because the distribution is complex according to the variables, and the effects of other variables are embedded. Therefore, this study examined the cause-effect relationship between obesity (variable) and fitness (multi-variable distribution) in the lump mean scheme (LMS) introduced by the present author to examine the obesity-dependence of the fitness test scores [9, 10] considering the body mass index (BMI) and waist-to-height ratio (WHT2R) as obesity indices. The WHT2R is a new waist-to-height ratio presented by the present author [10, 11].

The BMI is considered a useful index for assessing obesity because it is not intrusive and is easy to calculate with acceptable accuracy [9, 12–18]. The WHT2R is defined by the waist circumference divided by the square of the height [10] and was introduced because the waist circumference (WC) is more associated with cardio-metabolic mortality than the BMI, and height has an inverse association with mortality [19–22]. The WC has been considered an alternative anthropometric index that is more convenient and less expensive [23–30]. Moreover, the WHT2R is a more effective index for assessing obesity associated with fitness than the BMI and other waist-to-height ratios [10].

This study examined whether the cause-effect relations between fitness and BMI or WHT2R among Korean adults can be interpreted by their linearity and Gaussianity in the LMS. Linearity (or linear regression) and Gaussianity (or Gaussian regression) mean that the relationship between two variables are represented as a straight line and a Gaussian curve, respectively. The linear regression is a technique that is appropriate to understand the relation between an independent (cause) variable and a continuous dependent (effect) variable. An example of linear regression is the relation between total cholesterol (dependent variable) and body mass index (independent variable). This method cannot be applicable if a different relationship is hypothesized, such as a curvilinear or exponential relationship. The Gaussian regression is a principled, probabilistic approach to learn non-parametric models, where nonlinearity is implemented through kernels [31]. The Gaussian distribution is the most common distribution for random variables and fits many phenomena, such as blood pressure and height. The effect is determined by only one cause when a straight line represents the relationship and two or more causes when a Gaussian curve represents the relationship.

## Methods

### Lump mean scheme

**Fig. 1:**
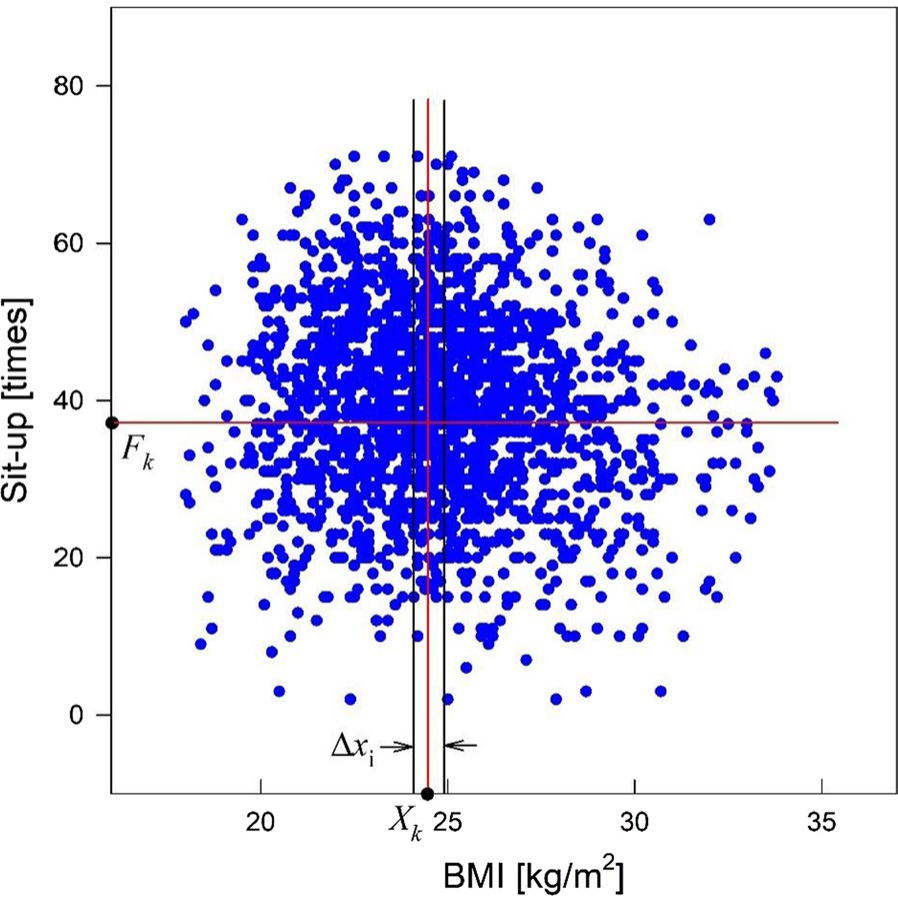
Males’ sit-up test scores with respect to BMIs

In many cases, it is almost impossible to investigate the dependence of a certain variable ${x}_{i}$ in a multi-variable distribution $f\left({x}_{i}, {y}_{i}, \dots \right)$. For example, Fig. 1 shows the distribution of males’ sit-up test scores ($f$) with respect to the BMI (${x}_{i}$) [9]. The BMI-dependence of the sit-up test score cannot be found from the data given in the figure, and it appears that the sit-up test score is affected by other factors, such as aging, muscle strength, or diseases. On the other hand, the sit-up scores around $\text{B}\text{M}\text{I}=24.5 [\text{k}\text{g}/{\text{m}}^{2}]$ (red vertical line) are relatively large and crowded. This indicates that the LMS can be considered.

To investigate a certain variable-dependence of multi-variable distribution, the lump mean values (LMVs), ${X}_{k}$ and ${F}_{k}$, of two variables ${x}_{i}$ and $f\left({x}_{i}, {y}_{i}, \dots \right)$ in $k$th lump with an interval of ${\varDelta x}_{i}$ were introduced, i.e., the LMS examined the characteristics of a multi-variable distribution by lumps. ${F}_{k}$ is independent of other variables because their effects are averaged out, and it can be clarified by choosing $\varDelta {x}_{i}\to 0$. This assumption is possible when the number of subjects is sufficiently large. Other variable-dependences of the distribution can be investigated in a similar manner.

### Linearity

The criteria for linearity are additivity and homogeneity, and they are satisfied by the equation for a straight line,

1 $$y\left(x\right)=mx$$

where $x$ and $y$ are two variables and $m$ is the slope. This study investigated the relationship using the equation,

2 $$y\left(x\right)=mx+c.$$

Equation (2) does not meet the criterion for the linearity by $c$ (y-intercept). Therefore, Eq. (2) means the linearity between the variations of $x$ and $y$, i.e., $dy=mdx$, which is the same form as Eq. (1) and $m$ determines the variation of $y$ for the variation of $x$. In this case, the effect ($y$) was determined by only one cause, i.e., $x$ includes only one cause.

### Gaussianity

In this study, the Gaussianity is satisfied when the distribution function $y\left(x\right)$ with respect to $x$ is expressed as a Gaussian distribution:

3 $$y\left(x\right)={y}_{0}+a\text{e}\text{x}\text{p}\left[-\frac{{\left(x-{x}_{0}\right)}^{2}}{2b}\right]$$

where ${y}_{0}$ is the minimum value of $y\left(x\right)$ when the constant $a$ is positive; $\sqrt{b}$ is the standard deviation, which determines the width of the distribution; ${x}_{0}$ is the $x$ value corresponding to the maximum value of $y\left(x\right)$. Differentiating Eq. (1) results in the following:

4 $$\frac{dy}{dx}=-\frac{a\left(x-{x}_{0}\right)}{b}\text{e}\text{x}\text{p}\left[-\frac{{\left(x-{x}_{0}\right)}^{2}}{2b}\right]$$

**Fig. 2:**
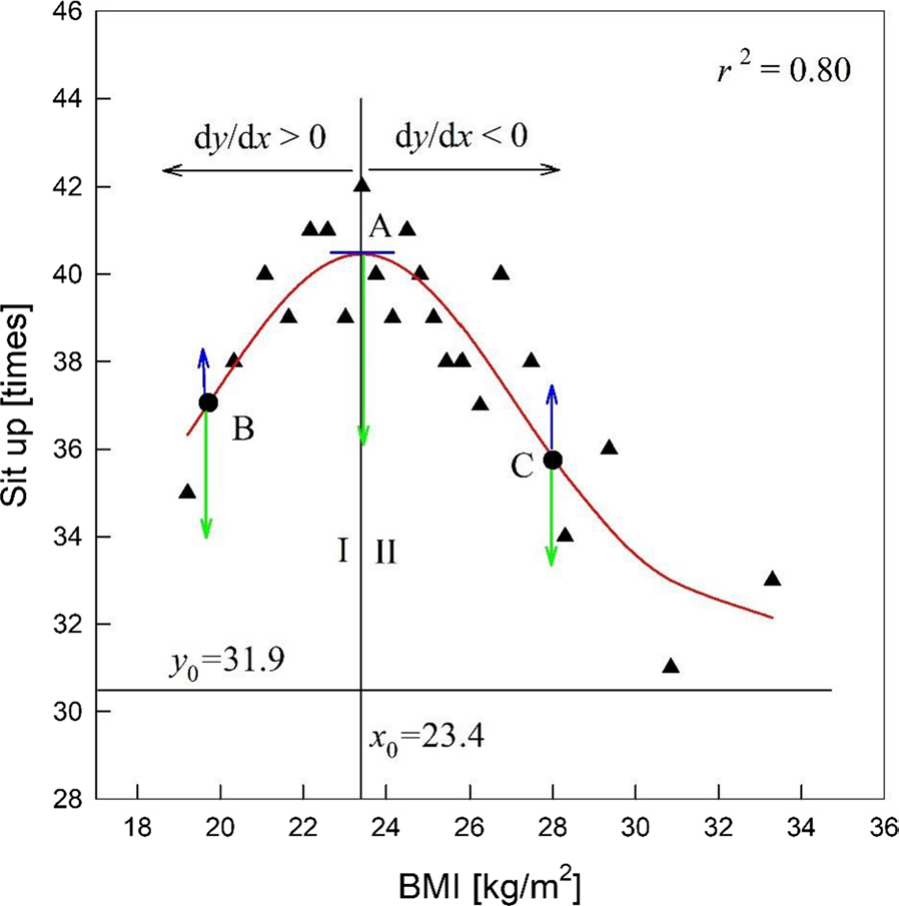
Black triangles are the males’ sit-up test scores for the BMI in the LMS, and the solid red curve denotes the curve for the black triangles fitted to Eq. (3) [9]. ${r}^{2}$ is the coefficient of determination

$y$ increases as $x$ increases when $x<{x}_{0}$ and $y$ decreases as $x$ increases when $x>{x}_{0}$ (see Fig. 2). Therefore, the effect ($y$) is determined by two causes, i.e., $x$ includes two causes.

### Newton mechanical interpretation

Differentiating Eq. (1) twice results in ${d}^{2}y/{dx}^{2}=0$. This is similar to the motion with constant acceleration, i.e., $x$ and $y$ correspond to time and position, respectively. In this case, an external force is absent, and the variation of $y$ with $x$ is constant.

Differentiating Eq. (3) twice results in the following:

5 $$b\frac{{d}^{2}y}{d{x}^{2}}=-\left(y-{y}_{0}\right)-\left(x-{x}_{0}\right)\frac{dy}{dx}={F}_{R}+{F}_{S}$$

Equation (5) is similar to the oscillatory motion with a damping term, i.e., the first term ${F}_{R}$ corresponds to the restoring force and the second term ${F}_{S}$ is the strengthening force that corresponds to the damping force in oscillatory motion.

In Eq. (5), $y$ has a property to decrease to its minimum value ${y}_{0}$ by the restoring force, $-\left(y-{y}_{0}\right),$ which increases with increasing $y-{y}_{0}$. $y$ is also increased by strengthening force, $-(x-{x}_{0})dy/dx$, which is always positive because $dy/dx\ge 0$ when $x-{x}_{0}\le 0$ and $dy/dx\le 0$ when $x-{x}_{0}\ge 0$ (Fig. 2). Therefore, $y$ tends to decrease by the restoring term and increase by the strengthening term because the restoring term is negative and the strengthening term is positive. The restoring term is natural because all things tend to restore to a stable state while the strengthening term forces $y$ to increase. The strengthening term can be interpreted by the law of inertia, i.e., $y$ has a property to prevent breaking away from its maximum value. Therefore, the Gaussian distribution is determined by competition of the restoring and strengthening terms and is represented by a curve with an extreme value, unlike straight lines.

### Design and analysis

This study was conducted using the results for the sit-up test among previous results [9, 10] obtained by the present author using the LMVs. The previous studies used the data extracted from the national physical fitness survey conducted in 2017 by the Korean Sports Promotion Foundation (KSPO) and approved by Statistics Korea (National Statistics No 113,004). The survey is a cross-sectional and nationally representative survey conducted periodically since 1989 to assess the fitness of Koreans. All of the participants provided informed consent prior to participation in the survey and the data were collected by age and gender using random allocation methods. The survey is publicly available from the KSPO. More details about study design and methods are provided on the KSPO website (http://www.sports.re.kr, available in Korean).

The sit-up test is a measure of muscular endurance [32]. The participants lie on the mat with knees bent at 90 degrees, feet flat on the floor and hands interlocked behind the head. The feet are held by a partner. The number of correctly performed sit-ups in one minute was recorded. The linearity or Gaussianity between the sit-up score and obesity were examined using Microsoft Excel 2014 and regressions in Sigmaplot 14. This study included 4296 Korean adults aged 19 to 64 years (2082 males and 2038 females) and excluded subjects who had extreme values of BMI (kg/m^2^), WHT2R ($\times {10}^{-4} {\text{c}\text{m}}^{-1}$) or scores on the sit-up (times/minute) test. The considered ranges as follows. For males, $18.0\le \text{B}\text{M}\text{I}\le 35.7$, $22.0\le \text{W}\text{H}\text{T}2\text{R}\le 39.9$ $2\le \text{S}\text{i}\text{t} \text{u}\text{p}\le 72$. For females, $16.0\le \text{B}\text{M}\text{I}\le 34.9$, $21.1\le \text{W}\text{H}\text{T}2\text{R}\le 43.6$ $2\le \text{S}\text{i}\text{t} \text{u}\text{p}\le 65$.

## Results

### Relationship between the sit-up test and BMI

Figure 2 shows that Gaussianity determines the males’ sit-up test score with respect to BMI in the LMS, where ${x}_{0}$ is the BMI for the maximum sit-up score. The data were well fitted to the Gaussian (red) curve because the coefficient of determination (${r}^{2}$) was sufficiently large.

The sit-up test score ($y$) increased with increasing BMI ($x$) until $x$ reached ${x}_{0}$ and then it decreased. This can be interpreted using Eq. (4) as follows. The variation of $y$ ($dy$) in region I is positive when $\text{d}x$ is positive because $x-{x}_{0}<0$, i.e., the sit-up score increases with increasing BMI. In region II, the sit-up score decreases with increasing BMI because $x-{x}_{0}>0$. This means that both fat and muscle included in the BMI are the dominant variables affecting the sit-up score (or muscular endurance), i.e., the sit-up score increases with increasing BMI in region I because the muscle increases and the sit-up score decreases with increasing BMI in region II because the fat increases. Therefore, muscle and fat are the causes, and the sit-up score is the effect.

In Fig. 2, the green down arrows and blue up arrows denote the restoring and strengthening terms, respectively. A larger difference means that more exercise is needed to maintain the score given by the curve. For example, subject A requires more exercise than subjects B and C to maintain the sit-up test score because the strengthening force of A is a minimum (zero), and its restoring force is a maximum. Therefore, the degradation of the sit-up score (or muscular endurance) of an athlete in good health (or high sit-up score) is large when they stop the exercise, and it is difficult to maintain good health. In Fig. 2, subject B in region I has a small muscle mass, hence weaker muscular endurance (or low sit-up score) than a subject with ${x}_{0}$. Moreover, subject C in region II has abundant fat and weaker muscular endurance than a subject with ${x}_{0}$.

If the subject B in region I increases their BMI using an exercise that increases muscle mass, the restoring term increases, and the strengthening term decreases. Hence, the sit-up score (muscular endurance) increases. On the other hand, if subject C in region II decreases their BMI using an exercise that decreases fat, the restoring term increases, and the strengthening term decreases, resulting in an increase in muscular endurance. The subjects above the curve have good health, i.e., they have more muscle and less fat than the subjects on the curve with the same BMI. In contrast, the subjects below the curve have poorer health than the subjects on the curve with the same BMI. Hence, they need to increase their muscle and decrese their fat by an exercise that increases their muscular endurance. Other fitness parameters, such as quickness, cardiorespiratory endurance, and speed and agility, can be investigated using the data for standing long jump, 20-m multistage shuttle run, and 10-m shuttle tests. The present method can also be applied to females [9].

### Relationship between the sit-up test and WHT2R

**Fig. 3:**
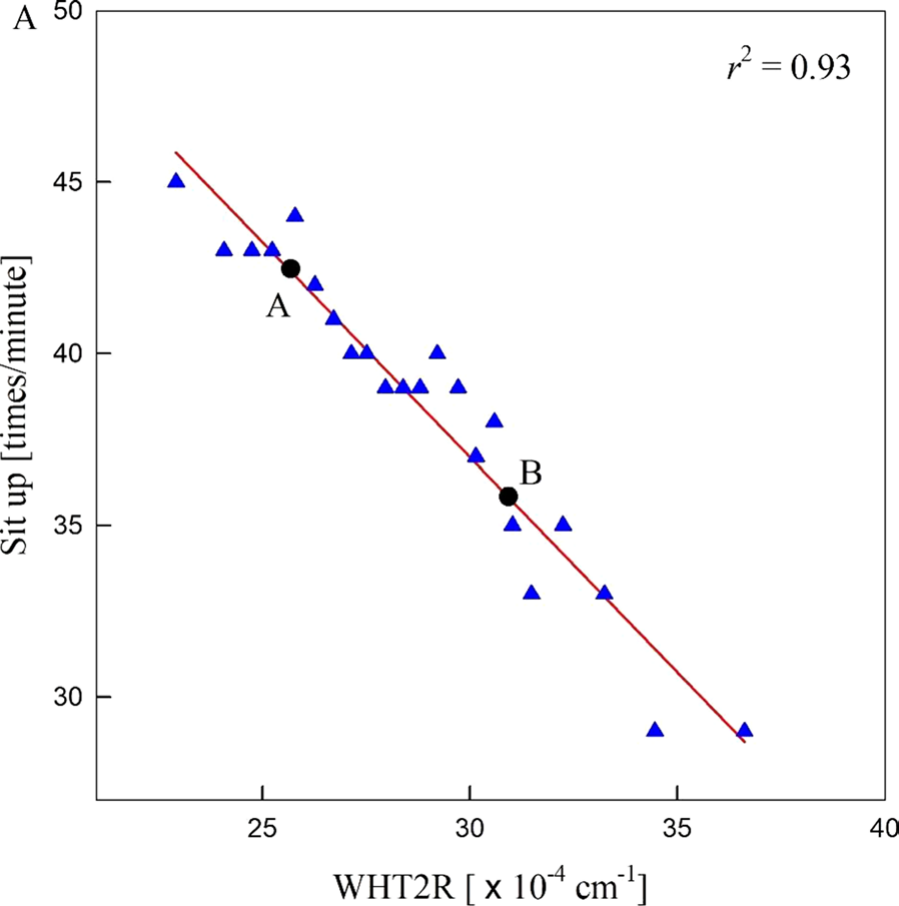
Blue triangles are the males’ sit-up test scores for WHT2R in the LMS, and the solid red straight line denotes the line for the blue triangles fitted to Eq. (2) [10]. ${r}^{2}$ is the coefficient of determination

Figure 3 shows that the males’ sit-up test score with respect to WHT2R in the LMS is determined by linearity, i.e., the sit-up test score decreases linearly with increasing WHT2R. Hence, the distribution of the sit-up test scores with respect to WHT2Rs is given as a straight line. The data were well fitted to the straight (red) line because the coefficient of determination (${r}^{2}$) is sufficiently large. This means that the abdominal fat included in WHT2R is only one dominant variable affecting the sit-up score (or muscular endurance). Therefore, the abdominal fat is the only cause, and the sit-up score is the effect. Setting the purpose of exercise using the WHT2R is simpler than using the BMI because the sit-up scores can be increased by an exercise that decreases the WHT2R. On the other hand, the BMI has merit in that it would be applicable to establish the exercise goal of people with poor fitness by controlling muscle and fat.

In Fig. 3, subject B has more abdominal fat and weaker muscular endurance (or low sit-up score) than subject A. Therefore, subject B needs to increase his muscular endurance by an exercise that decreases his abdominal fat. The subjects above (below) the line have better (poorer) health than the subjects on the line with the same WHT2R. Therefore, subjects above the line need to maintain their sit-up score for good health, and subjects below the line need to increase their muscular endurance by exercises that increase their sit-up score. Other fitness parameters can be investigated similarly, and the present method can be applied to females [10].

## Discussion

The relationship between fitness and BMI or WHT2R was investigated from the cause-effect relations using linearity and Gaussianity in the LMS. The distributions of male sit-up scores with respect to BMI and WHT2R are given as a Gaussian curve and straight line. Hussain et al. found the modest impact of BMI on lipid profile [33]. This paper found that there is also modest impact of BMI on fitness, i.e., the fitness increased to its maximum value with BMI and then decreased.

The personal exercise aims of Korean adults can be established from the present result. People below the Gaussian curve in region I of Fig. 2 can increase their muscular endurance by increasing their BMI using exercises that increase their muscle mass. In contrast, people below the Gaussian curve in region II can increase their muscular endurance by decreasing their BMI using exercises that decrease their fat. People below the straight line in Fig. 3 can increase their muscular endurance by decreasing their WHT2R using exercises that decrease their abdominal fat. Therefore, the WHT2R is a useful index to examine the cause-effect relationship between fitness and obesity. On the other hand, the BMI has merit in that it would be applicable to establish the exercise goal of people with poor fitness. Therefore, the WHT2R can be used as a more effective ratio to determine obesity and establish an exercise aim if the BMI is used in a complementary manner.

With the increasing mortality related to hypertension or diabetes, the necessity of effective methods to prevent them is increasing. The present method could be applied to examine the causes of hypertension or diabetes and to find a method to prevent them. In addition, the present method could be applied to females. Other fitness parameters, such as quickness, cardiorespiratory endurance, and speed and agility, can also be investigated. With the present method, effective exercises to increase fitness could be recommended individually, and the cause-effect relation of diseases, such as diabetes and hypertension, can be investigated. It is expected that the WHT2R can provide a more comprehensive indication of incident diabetes if it is combined with cardiorespiratory fitness [34].

## Conclusions

The relationship of muscular endurance with BMI and WHT2R could be interpreted from the cause-effect relations using Gaussianity and linearity, respectively. Male muscular endurance had two causes (fat and muscle) when BMI was selected as a variable and one cause (abdominal fat) when WHT2R was selected as a variable. This study showed that the WHT2R was a more convenient anthropometric index for predicting fitness than the BMI because the fitness with respect to the WHT2R could be fitted to a simple straight line while the fitness with respect to the BMI was fitted to the Gaussian curve [9]. Personal exercise aims were simpler to establish using WHT2R than using BMI. On the other hand, it was recommended for people with a low BMI to increase their fitness using exercises that increase their muscle mass.

## Data Availability

Data are available from the 2017 Survey of National Physical Fitness (ISBN 979-11-952035-6-7) conducted by the Korea Institute of Sport Science of the Korea Sports Promotion Foundation (KSPO) and are freely available from KSPO (https://www.kspo.or.kr).

## Abbreviations

LMS: Lump mean scheme
BMI: Body mass index
WHT2R: Waist-to-height ratio
WC: Waist circumference
